# Supplementary material for: Pyruvate Dehydrogenase Kinase 1 inhibition mediated oxidative phosphorylation enhancement in cartilage promotes osteoarthritis progression
Source: BMC Musculoskelet Disord. 2023 Jul 20;24:597. doi: 10.1186/s12891-023-06585-6 (PMC10357736; doi:10.1186/s12891-023-06585-6)
Supplement: Supplementary file 1 — Additional file 1. [file 12891_2023_6585_MOESM1_ESM.zip › BP.Gsea.1653623667859/GOBP_POSITIVE_REGULATION_OF_POSTTRANSCRIPTIONAL_GENE_SILENCING.html]

Details for gene set GOBP\_POSITIVE\_REGULATION\_OF\_POSTTRANSCRIPTIONAL\_GENE\_SILENCING[GSEA]

|  || Dataset | 55457\_collapsed\_to\_symbols.cls.cls #OA\_versus\_normal.cls.cls #OA\_versus\_normal\_repos |
| Phenotype | cls.cls#OA\_versus\_normal\_repos |
| Upregulated in class | normal |
| GeneSet | GOBP\_POSITIVE\_REGULATION\_OF\_POSTTRANSCRIPTIONAL\_GENE\_SILENCING |
| Enrichment Score (ES) | -0.51971585 |
| Normalized Enrichment Score (NES) | -1.7212969 |
| Nominal p-value | 0.0067453627 |
| FDR q-value | 0.120111674 |
| FWER p-Value | 1.0 |
Table: GSEA Results Summary

  

Fig 1: Enrichment plot: GOBP\_POSITIVE\_REGULATION\_OF\_POSTTRANSCRIPTIONAL\_GENE\_SILENCING      
 Profile of the Running ES Score & Positions of GeneSet Members on the Rank Ordered List

  

| SYMBOL | TITLE | RANK IN GENE LIST | RANK METRIC SCORE | RUNNING ES | CORE ENRICHMENT || 1 | EIF4E2 | eukaryotic translation initiation factor 4E family member 2 [Source:HGNC Symbol;Acc:HGNC:3293] | 1301 | 0.458 | -0.0553 | No |
| 2 | DDX6 | DEAD-box helicase 6 [Source:HGNC Symbol;Acc:HGNC:2747] | 1687 | 0.401 | -0.0453 | No |
| 3 | TGFB1 | transforming growth factor beta 1 [Source:HGNC Symbol;Acc:HGNC:11766] | 2490 | 0.303 | -0.0773 | No |
| 4 | FXR1 | FMR1 autosomal homolog 1 [Source:HGNC Symbol;Acc:HGNC:4023] | 3012 | 0.249 | -0.0929 | No |
| 5 | LIN28A | lin-28 homolog A [Source:HGNC Symbol;Acc:HGNC:15986] | 3140 | 0.236 | -0.0792 | No |
| 6 | MYCN | "MYCN proto-oncogene, bHLH transcription factor [Source:HGNC Symbol;Acc:HGNC:7559]" | 4554 | 0.109 | -0.1781 | No |
| 7 | EIF4ENIF1 | eukaryotic translation initiation factor 4E nuclear import factor 1 [Source:HGNC Symbol;Acc:HGNC:16687] | 5330 | 0.045 | -0.2338 | No |
| 8 | TP53 | tumor protein p53 [Source:HGNC Symbol;Acc:HGNC:11998] | 5511 | 0.032 | -0.2446 | No |
| 9 | PUM2 | pumilio RNA binding family member 2 [Source:HGNC Symbol;Acc:HGNC:14958] | 7186 | -0.095 | -0.3651 | No |
| 10 | LIMD1 | LIM domain containing 1 [Source:HGNC Symbol;Acc:HGNC:6612] | 7281 | -0.101 | -0.3623 | No |
| 11 | BMP4 | bone morphogenetic protein 4 [Source:HGNC Symbol;Acc:HGNC:1071] | 7816 | -0.142 | -0.3896 | No |
| 12 | FMR1 | FMRP translational regulator 1 [Source:HGNC Symbol;Acc:HGNC:3775] | 7944 | -0.151 | -0.3844 | No |
| 13 | STAT3 | signal transducer and activator of transcription 3 [Source:HGNC Symbol;Acc:HGNC:11364] | 8453 | -0.190 | -0.4049 | No |
| 14 | MAP2K2 | mitogen-activated protein kinase kinase 2 [Source:HGNC Symbol;Acc:HGNC:6842] | 9932 | -0.315 | -0.4883 | Yes |
| 15 | PUM1 | pumilio RNA binding family member 1 [Source:HGNC Symbol;Acc:HGNC:14957] | 10145 | -0.335 | -0.4713 | Yes |
| 16 | MAP2K1 | mitogen-activated protein kinase kinase 1 [Source:HGNC Symbol;Acc:HGNC:6840] | 10197 | -0.340 | -0.4413 | Yes |
| 17 | DHX9 | DExH-box helicase 9 [Source:HGNC Symbol;Acc:HGNC:2750] | 10627 | -0.386 | -0.4361 | Yes |
| 18 | TIAL1 | TIA1 cytotoxic granule associated RNA binding protein like 1 [Source:HGNC Symbol;Acc:HGNC:11804] | 11049 | -0.438 | -0.4252 | Yes |
| 19 | AGO2 | argonaute RISC catalytic component 2 [Source:HGNC Symbol;Acc:HGNC:3263] | 11246 | -0.463 | -0.3942 | Yes |
| 20 | TNRC6B | trinucleotide repeat containing adaptor 6B [Source:HGNC Symbol;Acc:HGNC:29190] | 11947 | -0.582 | -0.3905 | Yes |
| 21 | RIPK1 | receptor interacting serine/threonine kinase 1 [Source:HGNC Symbol;Acc:HGNC:10019] | 11953 | -0.584 | -0.3326 | Yes |
| 22 | EGFR | epidermal growth factor receptor [Source:HGNC Symbol;Acc:HGNC:3236] | 12109 | -0.622 | -0.2825 | Yes |
| 23 | EIF4G1 | eukaryotic translation initiation factor 4 gamma 1 [Source:HGNC Symbol;Acc:HGNC:3296] | 12122 | -0.625 | -0.2211 | Yes |
| 24 | IL6 | interleukin 6 [Source:HGNC Symbol;Acc:HGNC:6018] | 12460 | -0.730 | -0.1745 | Yes |
| 25 | DDX5 | DEAD-box helicase 5 [Source:HGNC Symbol;Acc:HGNC:2746] | 12834 | -1.038 | -0.0999 | Yes |
| 26 | ZFP36 | ZFP36 ring finger protein [Source:HGNC Symbol;Acc:HGNC:12862] | 12846 | -1.052 | 0.0042 | Yes |
Table: GSEA details [plain text format]

  

Fig 2: GOBP\_POSITIVE\_REGULATION\_OF\_POSTTRANSCRIPTIONAL\_GENE\_SILENCING      
 Blue-Pink O' Gram in the Space of the Analyzed GeneSet

  

Fig 3: GOBP\_POSITIVE\_REGULATION\_OF\_POSTTRANSCRIPTIONAL\_GENE\_SILENCING: Random ES distribution      
 Gene set null distribution of ES for **GOBP\_POSITIVE\_REGULATION\_OF\_POSTTRANSCRIPTIONAL\_GENE\_SILENCING**
